# Draft Genome Sequence of *Photorhabdus luminescens* HIM3 Isolated from an Entomopathogenic Nematode in Agricultural Soils

**DOI:** 10.1128/genomeA.00745-17

**Published:** 2017-08-31

**Authors:** Rosalba Salgado-Morales, Nancy Rivera-Gómez, Fernando Martínez-Ocampo, Luis Fernando Lozano-Aguirre Beltrán, Armando Hernández-Mendoza, Edgar Dantán-González

**Affiliations:** a Laboratorio de Estudios Ecogenómicos, Centro de Investigación en Biotecnología, Universidad Autónoma del Estado de Morelos, Cuernavaca, Morelos, Mexico; b Centro de Ciencias Genómicas, Programa de Genómica Evolutiva, Universidad Nacional Autónoma de México, Cuernavaca, Morelos, Mexico; c Centro de Investigación en Dinámica Celular, Universidad Autónoma del Estado de Morelos, Cuernavaca, Morelos, Mexico

## Abstract

In this work, we report the draft genome sequence of *Photorhabdus luminescens* strain HIM3, a symbiotic bacterium associated with the entomopathogenic nematode *Heterorhabditis indica* MOR03, isolated from soil sugarcane in Yautepec, Morelos, Mexico. These bacteria have a G+C content of 42.6% and genome size of 5.47 Mb.

## GENOME ANNOUNCEMENT

The bacterium *Photorhabdus luminescens* is a gammaproteobacterium, is Gram negative, and is a member of the *Enterobacteriaceae* family; it is highly lethal for a wide spectrum of insects and maintains a symbiotic relationship with nematodes of the genus *Heterorhabditis* (1). The nematode carries the bacterium in the intestine during the juvenile infective stage (IJ), which penetrates an insect and regurgitates the symbionts in the hemolymph; these bacteria kill the insect in 24 to 48 h (2, 3). Therefore, *P. luminescens* is a good model for understanding the genetic bases involved in mutualism with nematodes and pathogenicity against insects, as well as being a source of insecticidal toxins (4, 5).

We sequenced the genome of the bacterium *P. luminescens* strain HIM3, isolated from the entomopathogenic nematode *Heterorhabditis indica* MOR03, recovered from soil cultivated with sugarcane in Yautepec, Morelos, Mexico (18°55′16.9″N, 99°02′24.0″W). The bacteria were grown in LB broth and incubated for 12 h at 30°C with shaking at 250 rpm; genomic DNA was extracted with the ZR fungal/bacterial miniprep kit (Zymo Research), and 5 µg of genomic DNA was sequenced using the Illumina HiSeq platform (2 × 300 bp paired-end reads). A total of 814,835 reads were obtained, and quality trimming and error correction of the reads were performed using a DynamicTrim (SolexaQA++) (6) Perl script. Reads were assembled using SPAdes version 3.5.0 (7), and the draft genome resulted in 91 contigs with a total length of 5,472,253 bp. The largest contig was 706,123 bp, the *N*_50_ value was 232,526 bp, and the *L*_50_ was reached in 8 contigs. The genome sequence of *P. luminescens* subsp.* laumondii* TTO1 was used as a reference. Automated annotation was performed with Rapid Annotations using Subsystems Technology (RAST) (8). The genome of *P. luminescens* HIM3 has a G+C content of 42.6% and includes 4,659 coding sequences; using RNAmmer and Aragorn, 10 rRNAs (5S, 16S, and 23S) and 70 tRNAs, respectively, were obtained (9, 10). Based on the analysis of concatenated sequences (*gyrA, gyrB, dnaN*, and 16S and 23S rRNA genes) *P. luminescens* HIM3 is closely related to *P. luminescens* DSM 3368 (GenBank accession no. NZ_JXSK00000000). The genome of *P. luminescens* HIM3 contains genes encoding the type III secretion system (T3SS) responsible for the injection of virulence proteins into the cytoplasm of eukaryotic cells, and it contains the gene coding for YopT, an effector protein that inhibits phagocytosis in insect macrophages (11). Interestingly, besides pathogenesis, it is suggested that T3SS is involved in other symbiotic relationships, such as mutualism and parasitism (12). We also found genes encoding the repeats-in toxin (RTX)-like toxins, including PrtA, a metalloprotease secreted by the type I secretion system (T1SS), which has been proposed to be involved in degrading insect tissues, which are food sources of nematode and bacteria (13). Also, genes coding for multiple type II toxin-antitoxin modules and colicin-like bacteriocins that prevent the growth of nonsymbiotic bacteria and insect putrefaction were found. The presence of plasmids was not found using PlasmidFinder 1.3 (14). We found genes necessary for the efficient uptake of iron, an important factor for pathogenicity and mutualism (15). Finally, large numbers of genes with probable insecticidal activity were found in the genome of *P. luminescens* HIM3.

### Accession number(s).

This whole-genome shotgun project has been deposited at DDBJ/ENA/GenBank under the accession no. MYFJ00000000. The version described in this paper is version MYFJ01000000.
